# Supplementary material for: The Arf-GAP Age2 localizes to the late-Golgi via a conserved amphipathic helix
Source: bioRxiv. 2023 Jul 24:2023.07.23.550229. Preprint. [Version 1] doi: 10.1101/2023.07.23.550229 (PMC10402032; doi:10.1101/2023.07.23.550229)
Supplement: 1 [file NIHPP2023.07.23.550229V1-supplement-1.pdf]

bioRxiv preprint doi: https://doi.org/10.1101/2023.07.23.550229; this version posted July 24, 2023. The copyright holder for this preprint (which was not certified by peer review) is the author/funder, who has granted bioRxiv a license to display the preprint in perpetuity. It is made available under aCC-BY 4.0 International license.

Supplemental Figures

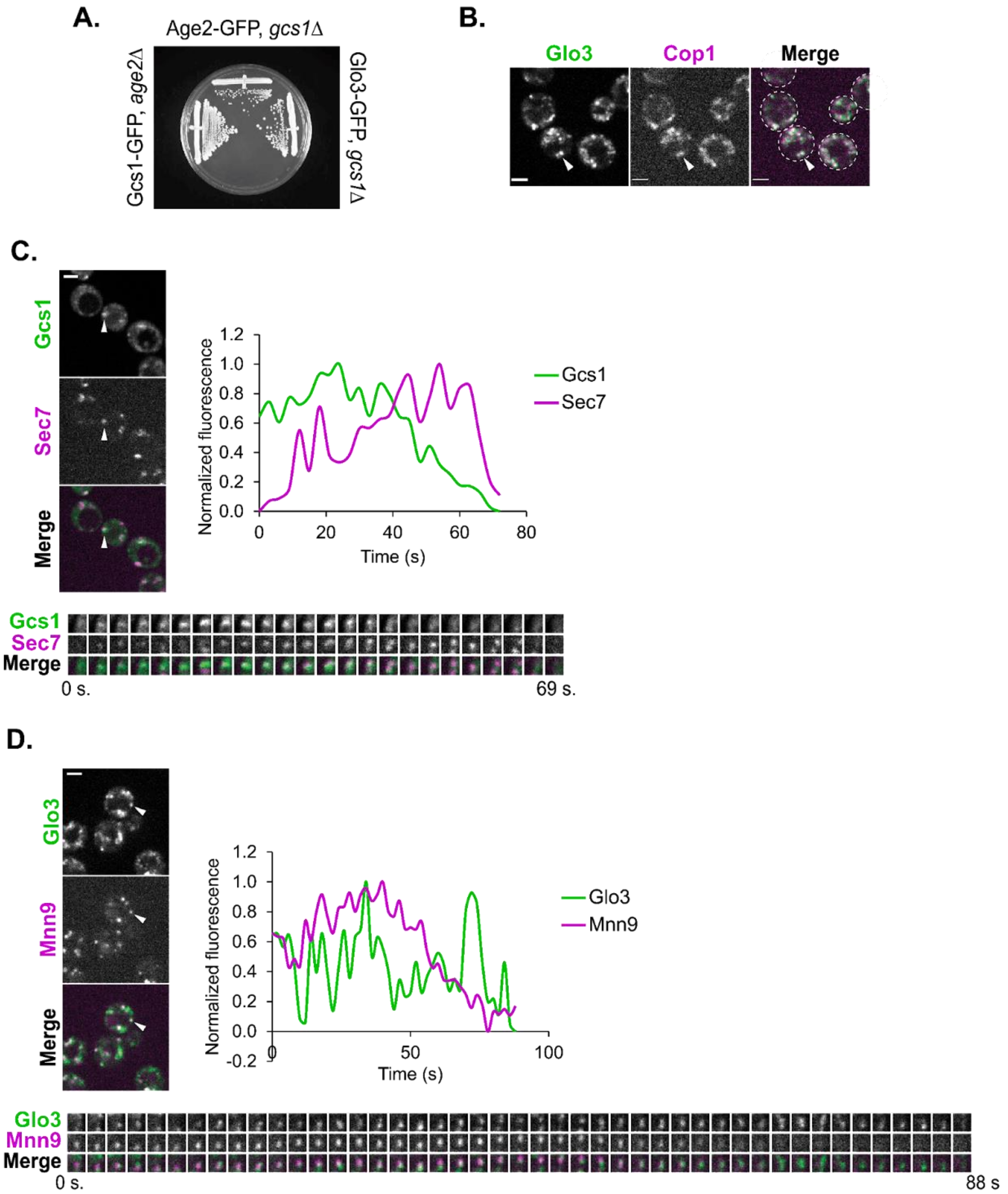

Supplemental Figure 1. Timelapse analyses of Arf-GAP maturation dynamics at the Golgi.
(A) Growth assay of tagged Arf-GAPs in strains lacking another Arf-GAP that together form an essential pair. (B) Subcellular localization of Glo3-Neon relative to the COPI subunit, Cop1-mCherry. (C) Left: Representative image of time-lapse microscopy of Gcs1-GFP versus Sec7-6xDsRed. Arrowhead denotes Golgi compartment of interest. Bottom: imaging of the compartment of interest over time. Right: plot of normalized fluorescence intensity in the compartment of interest over time. (D) Left: Representative image of time-lapse microscopy of Glo3-Neon versus Mnn9-mCherry. Arrowhead denotes Golgi compartment of interest. Bottom: imaging of the compartment of interest over time. Top right: plot of normalized fluorescence intensity in the compartment of interest over time.

bioRxiv preprint doi: https://doi.org/10.1101/2023.07.23.550229; this version posted July 24, 2023. The copyright holder for this preprint (which was not certified by peer review) is the author/funder, who has granted bioRxiv a license to display the preprint in perpetuity. It is made available under aCC-BY 4.0 International license.

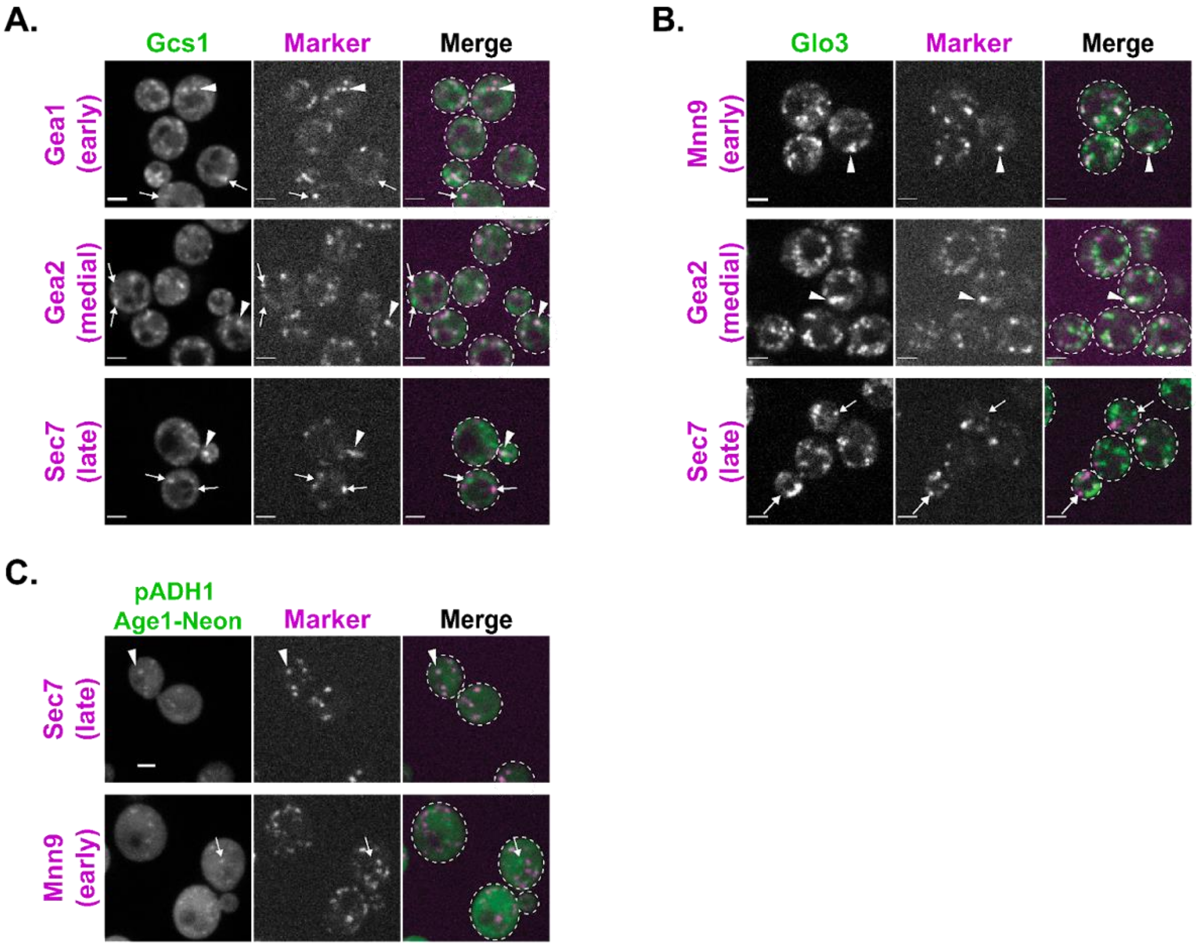

Supplemental Figure 2. Colocalization analysis of Arf-GAPs and Golgi compartment markers. (A) Subcellular localization of Gcs1 relative to early- (Gea1-3xMars), medial- (Gea2-3xMars), or late- (Sec7-6xDsRed) Golgi markers. Gcs1 is tagged with GFP in the Gea1-3xMars strain, and Neon in the Gea2-3xMars and Sec7-6xDsRed strain. Arrowheads and arrows indicate colocalization or a lack of colocalization between proteins, respectively. Single focal planes. (B) Subcellular localization of Glo3 relative to early- (Mnn9-mCherry), medial- (Gea2-3xMars), or late- (Sec7-6xDsRed) Golgi markers. Glo3 is tagged with GFP in the Sec7-6xDsRed strain, and Neon in all other strains. Arrowheads and arrows indicate either colocalization or a lack of colocalization between proteins, respectively. Single focal planes. (C) Subcellular localization of Age1 overexpressed on a centromeric plasmid under the strong ADH1 promoter relative to markers of the late- (Sec7-6xDsRed) or early- (Mnn9-mCherry) Golgi. Maximum projections. For all images, scale bars represent 2 µm.

bioRxiv preprint doi: https://doi.org/10.1101/2023.07.23.550229; this version posted July 24, 2023. The copyright holder for this preprint (which was not certified by peer review) is the author/funder, who has granted bioRxiv a license to display the preprint in perpetuity. It is made available under aCC-BY 4.0 International license.

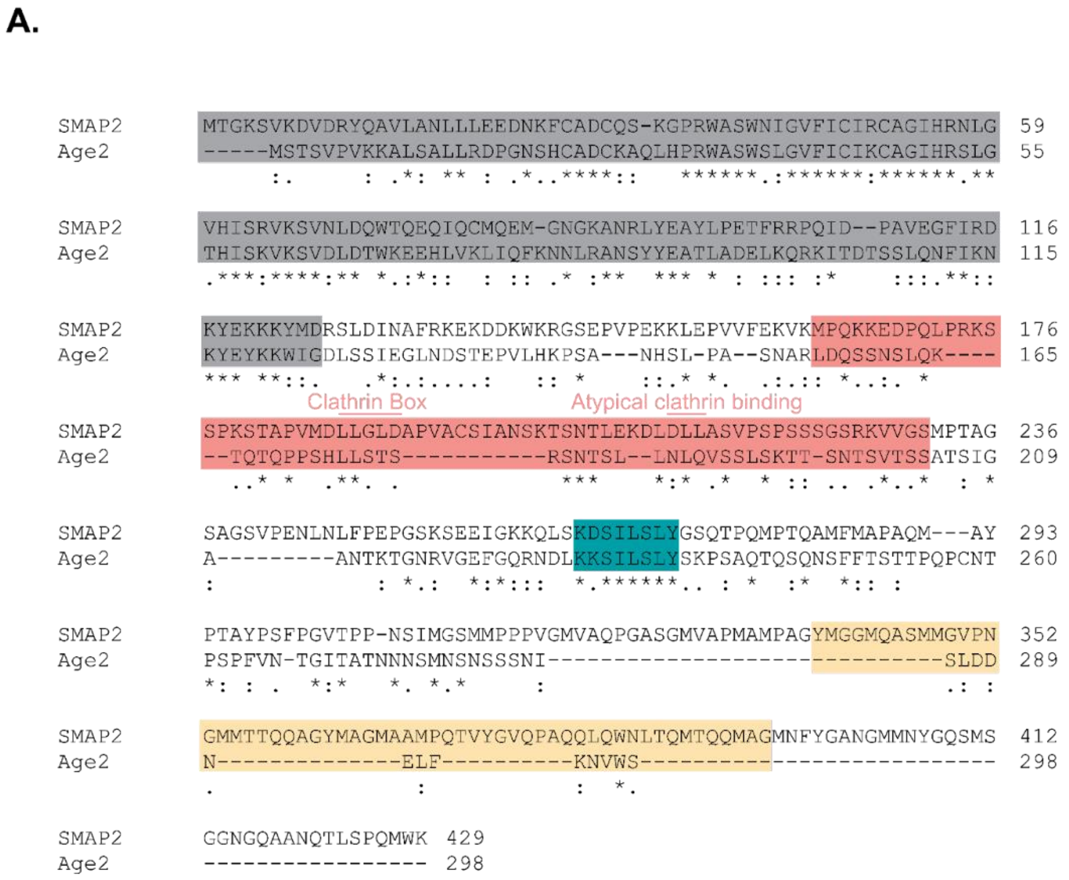

Supplemental Figure 3. Alignment of Age2 and SMAP2.
(A) Alignment of Age2 and SMAP2 (accession numbers P40529 and Q8WU79 respectively). SMAP2 functional domains (Natsume et al., 2006) are highlighted (GAP domain in gray, clathrin interaction domain in pink, and CALM interaction domain in yellow). The conserved KKSILSLY sequence is also highlighted in teal. The clathrin interacting motifs within the clathrin interaction domain are denoted.

bioRxiv preprint doi: https://doi.org/10.1101/2023.07.23.550229; this version posted July 24, 2023. The copyright holder for this preprint (which was not certified by peer review) is the author/funder, who has granted bioRxiv a license to display the preprint in perpetuity. It is made available under aCC-BY 4.0 International license.

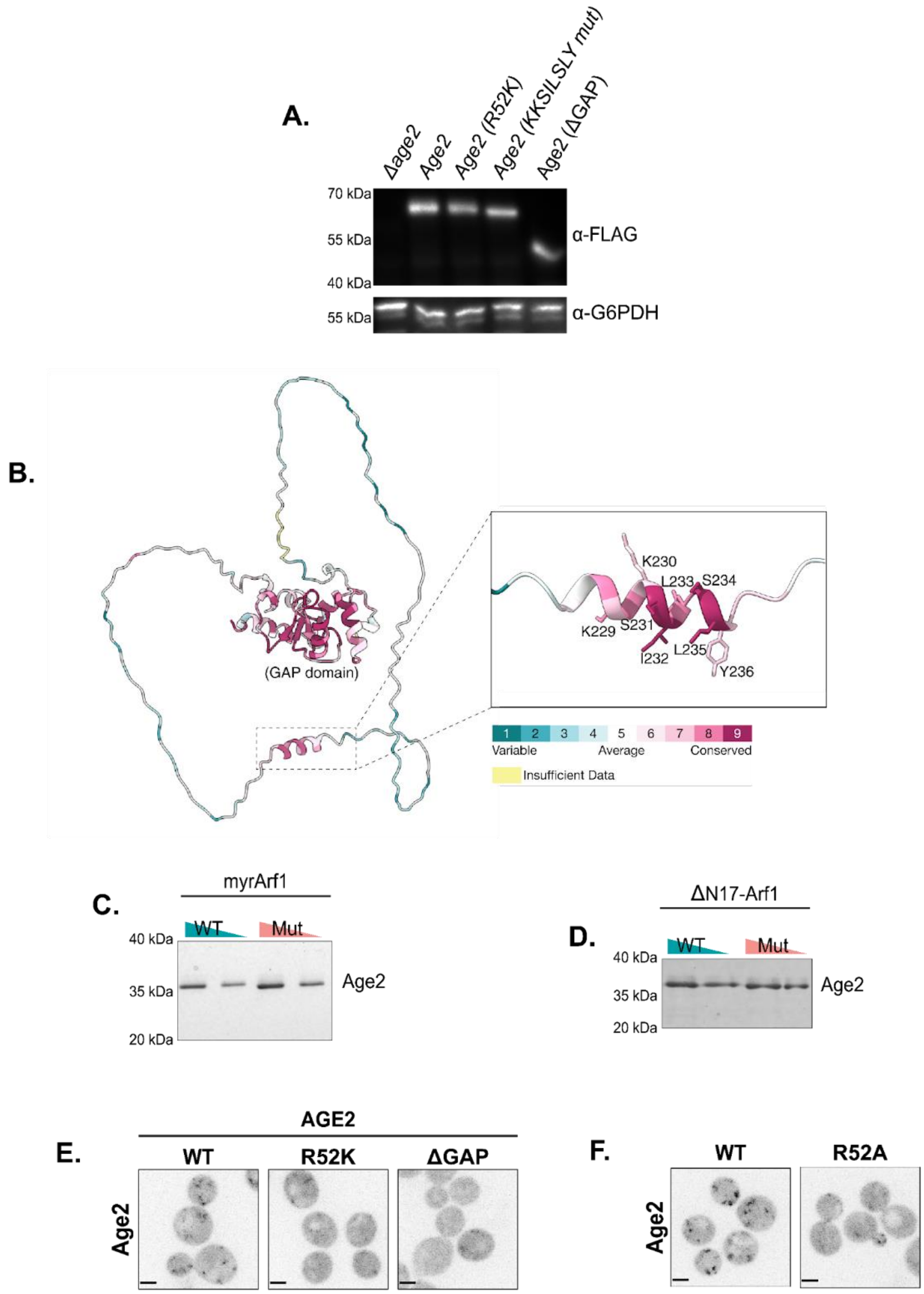

Supplemental Figure 4. Further validation of the dependency of Age2 on Arf1 and membranes for localization. (A) Western blot analysis of whole cell lysates expressing Age2-Neon-3xFLAG mutants using anti-FLAG. Glucose-6-Phosphate Dehydrogenase (G6PDH) serves as a loading control. (B) AlphaFold prediction of Age2 structure, with a close up of the predicted helix formed by the conserved KKSILSLY sequence (Jumper et al. 2021; Varadi et al. 2021). Model is colored by conservation as determined by ConSurf (Glaser et al., 2003; Landau et al., 2005). (C) SDS-PAGE of purified proteins used for GAP assays with myrArf1. Triangles indicate the relative amount of protein loaded. (D) SDS-PAGE of purified protein used for GAP assays with ΔN17-Arf1. Triangles indicate the relative amount of protein loaded. (E) Fluorescence microscopy of Age2-Neon-3xFLAG mutants expressed as an extra copy in an otherwise wild-type *AGE2* strain. Maximum projections. (F) Fluorescence microscopy of GAP dead Age2(R52A)-Neon-3xFLAG. Maximum projections. For all images, scale bars represent 2 µm.

bioRxiv preprint doi: https://doi.org/10.1101/2023.07.23.550229; this version posted July 24, 2023. The copyright holder for this preprint (which was not certified by peer review) is the author/funder, who has granted bioRxiv a license to display the preprint in perpetuity. It is made available under aCC-BY 4.0 International license.

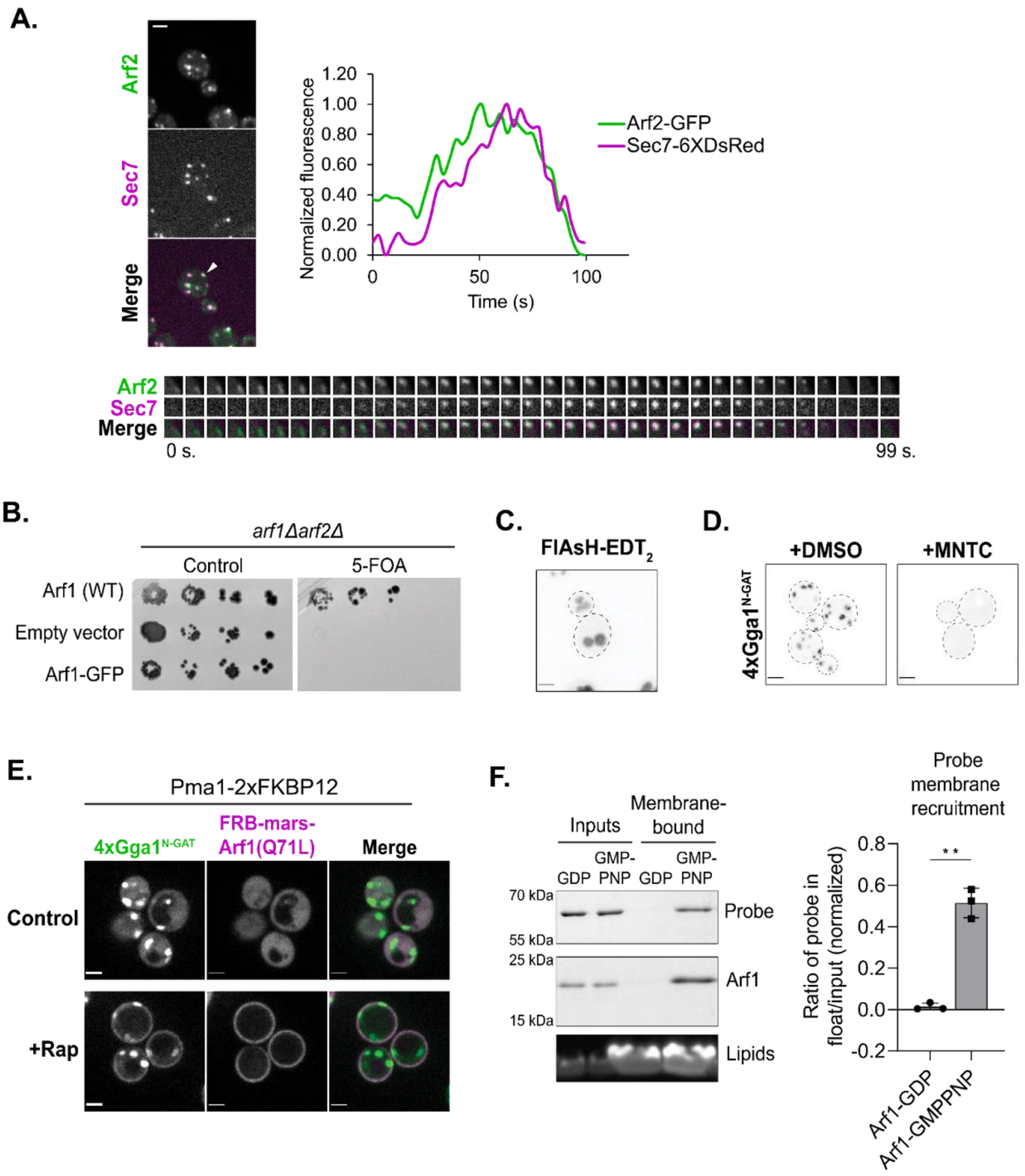

Supplemental Figure 5. Validation of FlAsH-labeling approach and 4xGga1$^{N\text{-}GAT}$-Neon probe.
(A) Left: Representative image of time-lapse microscopy of Arf2-GFP versus Sec7-6xDsRed. Arrowhead denotes Golgi compartment of interest. Bottom: Imaging of the compartment of interest over time. Top right: Plot of normalized fluorescence intensity in the compartment of interest over time. (B) Complementation assay with Arf1 tagged with GFP. (C) Representative image of FlAsH-treated wild-type cells showing vacuolar staining. Maximum projection. (D) Fluorescence microscopy of 4xGga1$^{N\text{-}GAT}$-Neon after treatment with either MNTC or DMSO (control) for 1 minute. (E) Representative images of 4xGga1$^{N\text{-}GAT}$-Neon in cells expressing FKBP-tagged Pma1 and FRB-tagged GTP-locked (Q71L) Arf1 treated with rapamycin to induce dimerization of FKBP and FRB and thus the relocalization of Arf1 to the plasma membrane. Single focal plane. (F) Left: *In vitro* liposome flotation assay to assess binding of purified protein to membranes in an Arf1-dependent manner. Right: Quantification of 4xGga1$^{N\text{-}GAT}$-Neon probe membrane recruitment, as measured by coomassie band intensity ratio. Error bars represent standard deviation for n = 3 assays.

bioRxiv preprint doi: https://doi.org/10.1101/2023.07.23.550229; this version posted July 24, 2023. The copyright holder for this preprint (which was not certified by peer review) is the author/funder, who has granted bioRxiv a license to display the preprint in perpetuity. It is made available under aCC-BY 4.0 International license.

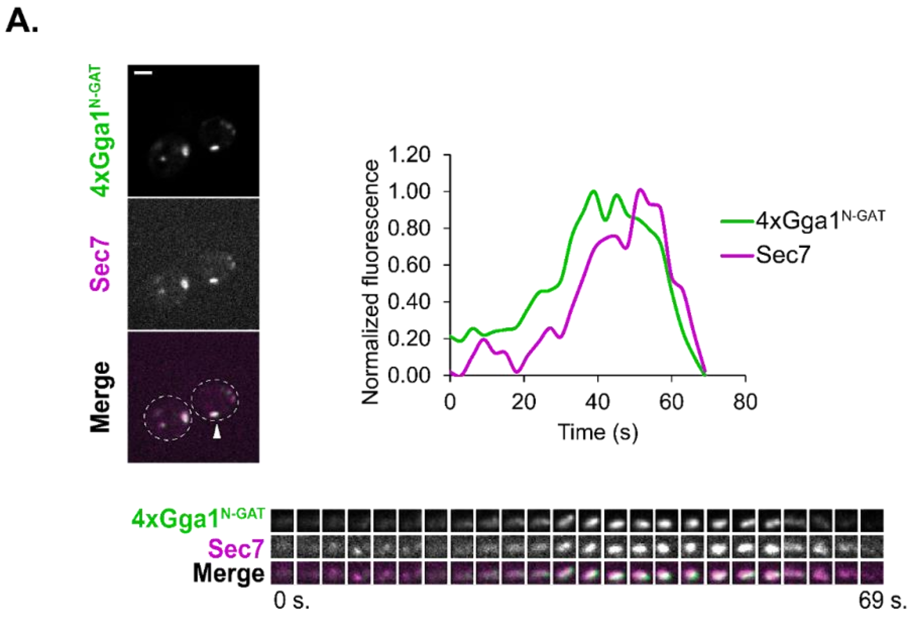

Supplemental Figure 6. Time-lapse analysis of 4xGga1$^{N\text{-}GAT}$ maturation dynamics.
(A) Left: Representative image of time-lapse microscopy of 4xGga1$^{N\text{-}GAT}$-Neon versus Sec7-6xDsRed. Arrowhead denotes Golgi compartment of interest. Bottom: Imaging of the compartment of interest over time. Top right: Plot of normalized fluorescence intensity in the compartment of interest over time.

bioRxiv preprint doi: https://doi.org/10.1101/2023.07.23.550229; this version posted July 24, 2023. The copyright holder for this preprint (which was not certified by peer review) is the author/funder, who has granted bioRxiv a license to display the preprint in perpetuity. It is made available under aCC-BY 4.0 International license.

Table S1. Yeast strains used in this study:

Strain	Description	Source
**SEY6210**	*MATα ura3-52 his3-Δ200 leu2-3,112 lys2-801 trp1-Δ901 suc2-Δ9*	(J. S. Robinson et al., 1988)
**SEY6210.1**	*MATa ura3-52 his3-Δ200 leu2-3,112 lys2-801 trp1-Δ901 suc2-Δ9*	(J. S. Robinson et al., 1988)
**BJ5459**	*MATa pep4::HIS3 prbΔ1.6R can1*	(Hickey et al., 2009; Jones, 1991)
**BY4741a**	*MATa ura3-Δ0 his3-Δ1 leu2-Δ0 met15-Δ0*	(Brachmann et al., 1998)
**CFY564**	SEY6210.1 *age2Δ::HIS3 gcs1Δ::KANMX [pRS416 Age2] (pCF1066)*	This study
**CFY602**	SEY6210 *arf1Δ::His3 arf2Δ::KanMX Sec7-Mars::TRP1 [pRS416 Arf1] (pCF1022)*	This study
**CFY1689**	SEY6210.1 *Sec7-6xDsRed::URA3::ura3*	(McDonold & Fromme, 2014)
**CFY2277**	SEY6210.1 *Sec7-6xDsRed::URA3 Arf2-GFP::HIS3*	This study
**CFY4138**	SEY6210.1 *Sec7-6xDsRed::URA3::ura3 Age2-GFP::HIS3*	This study
**CFY4187**	SEY6210.1 *Sec7-6xDsRed::URA3::ura3 Glo3-GFP::HIS3*	This study
**CFY4190**	SEY6210.1 *Sec7-6xDsRed::URA3::ura3 Gcs1-GFP::HIS3*	This study
**CFY4243**	SEY6210.1 *Cop1-mCherry::KanMX Glo3-Neon::HIS3*	This study
**CFY4267**	SEY6210.1 Sec7-6xDsRed::URA3::ura3 Glo3-GFP::HIS3 *gcs1Δ::KanMX*	This study
**CFY4271**	SEY6210.1 *Sec7-6xDsRed::URA3::ura3 Arf1-TetCys::HIS3*	This study
**CFY4275**	SEY6210 *Sec7-6xDsRed::URA3::ura3 Age2-GFP::HIS3 gcs1Δ::KanMX*	This study
**CFY4290**	SEY6210.1 *Sec7-6xDsRed::URA3::ura3 Gcs1-GFP::HIS3 age2Δ::KanMX*	This study
**CFY4296**	SEY6210.1 *Gea1-3xMars::TRP1 Gcs1-Neon::HIS3*	This study

bioRxiv preprint doi: https://doi.org/10.1101/2023.07.23.550229; this version posted July 24, 2023. The copyright holder for this preprint (which was not certified by peer review) is the author/funder, who has granted bioRxiv a license to display the preprint in perpetuity. It is made available under aCC-BY 4.0 International license.

**CFY4299**	SEY6210.1 *Gea2-3xMars::TRP1 Gcs1-Neon:HIS3*	This study
**CFY4304**	SEY6210.1 *Sec7-6xDsRed::URA3::ura3 Age1-Neon::HIS3*	This study
**CFY4305**	SEY6210 *Gcs1-Neon::HIS3 Sec7-6xDsRed::URA3*	This study
**CFY4333**	SEY6210.1 *Sec7-6xDsRed::URA3::ura3 leu2::4xGga1*$^{N\text{-}GAT}$*-Neon::LEU2*	This study
**CFY4349**	W303 *tor1-1 fpr1::NatMX Pma1-2xFKBP12::TRP1 leu2::4xGga1*$^{N\text{-}GAT}$*-Neon::LEU2*	This study
**CFY4357**	SEY6210 *Mnn9-mCherry::HIS3*	(Sardana et al., 2021)
**CFY4372**	SEY6210 *Mnn9-mCherry::HIS3 Glo3-Neon-3XHA::TRP1*	This study
**CFY4414**	SEY6210.1 *Sec7-6xDsRed::URA3 age2Δ::KanMX*	This study
**CFY4452**	SEY6210.1 *Gea1-3xMars::TRP1 Arf1-TetCys::HIS3*	This study
**CFY4454**	SEY6210.1 *Gea2-3xMars::TRP1 Arf1-TetCys::HIS3*	This study
**CFY4484**	BY4741a *apl5Δ::KanMX*	ResGen
**CFY4485**	BY4741a *chc1Δ::KanMX*	ResGen
**CFY4510**	SEY6210.1 *apl2Δ::KANMX Sec7-MARS::TRP1 Age2-Neon::HIS3*	This study
**CFY4535**	SEY6210 *gga1Δ::TRP1 gga2Δ::HIS3 Sec7-6xDsRed::URA3::ura3 Age2-Neon::LEU2*	This study
**CFY4554**	SEY6210.1 *age2Δ::KanMX Sec7-6xDsRed::URA3 leu2::Age2-Neon-3xFLAG::LEU2*	This study
**CFY4557**	SEY6210.1 *age2Δ::KanMX Sec7-6xDsRed::URA3 leu2::Age2(S231A)-Neon-3xFLAG::LEU2*	This study
**CFY4558**	SEY6210.1 *age2Δ::KanMX Sec7-6xDsRed::URA3 leu2::Age2(I232A)-Neon-3xFLAG::LEU2*	This study
**CFY4560**	SEY6210.1 *age2Δ::KanMX Sec7-6xDsRed::URA3 leu2::Age2(L233A)-Neon-3xFLAG::LEU2*	This study
**CFY4562**	SEY6210.1 *age2Δ::KanMX Sec7-6xDsRed::URA3 leu2::Age2(S234A)-Neon-3xFLAG::LEU2*	This study

bioRxiv preprint doi: https://doi.org/10.1101/2023.07.23.550229; this version posted July 24, 2023. The copyright holder for this preprint (which was not certified by peer review) is the author/funder, who has granted bioRxiv a license to display the preprint in perpetuity. It is made available under aCC-BY 4.0 International license.

**CFY4565**	SEY6210.1 *age2Δ::KanMX Sec7-6xDsRed::URA3 leu2::Age2(L235A)-Neon-3xFLAG::LEU2*	This study
**CFY4566**	SEY6210.1 *age2Δ::KanMX Sec7-6xDsRed::URA3 leu2::Age2(Y236A)-Neon-3xFLAG::LEU2*	This study
**CFY4569**	SEY6210.1 *age2Δ::KanMX Sec7-6xDsRed::URA3 leu2::Age2(KK229AA)-Neon-3xFLAG::LEU2*	This study
**CFY4622**	SEY6210 *gga1Δ::TRP1 gga2Δ::HIS3 apl2Δ::KANMX Sec7-6xDsRed::URA3::ura3 Age2-Neon::LEU2*	This study
**CFY4663**	SEY6210.1 *Gea2-3xMars::TRP1 Glo3-Neon::HIS3*	This study
**CFY4697**	SEY6210.1 *age2Δ::KanMX Sec7-6xDsRed::URA3 leu2::Age2(R52K)-Neon-3xFLAG::LEU2*	This study
**CFY4761**	BJ5459 *pep4::HIS3 prbΔ1.6R can1 pYC230 GST(PreScission) Age2 (GAL1 promoter)*	This study
**CFY4810**	SEY6210.1 *age2Δ::KanMX Sec7-6xDsRed::URA3 leu2::Age2-Neon-3xFLAG::LEU2*	This study
**CFY4813**	SEY6210.1 *age2Δ::KanMX Sec7-6xDsRed::URA3 leu2::Age2(M1-P239)-Neon-3xFLAG::LEU2*	This study
**CFY4815**	SEY6210.1 *age2Δ::KanMX Sec7-6xDsRed::URA3 leu2::Age2*(M1-P150)-GS-Age2(G223-S298)*-Neon-3xFLAG::LEU2*	This study
**CFY4817**	SEY6210.1 *age2Δ::KanMX Sec7-6xDsRed::URA3 leu2::Age2(I129 - S298)-Neon-3xFLAG::LEU2*	This study
**CFY4818**	SEY6210.1 *age2Δ::KanMX Sec7-6xDsRed::URA3 leu2::Gcs1(M1-H132)-Age2(I129-S298)-Neon-3xFLAG::LEU2*	This study
**CFY4857**	BJ5459 *pep4::HIS3 prbΔ1.6R can1 pYC230 GST(PreScission) Age2 (KKSILSLY 229 AAAAAAAA) (GAL1 promoter)*	This study
**CFY4875**	SEY6210.1 *Sec7-6xDsRed::URA3::ura3 leu2::Age2-Neon-3xFLAG::LEU2*	This study
**CFY4877**	SEY6210.1 *Sec7-6xDsRed::URA3::ura3 leu2::Age2 (R52K)-Neon-3xFLAG::LEU2*	This study

bioRxiv preprint doi: https://doi.org/10.1101/2023.07.23.550229; this version posted July 24, 2023. The copyright holder for this preprint (which was not certified by peer review) is the author/funder, who has granted bioRxiv a license to display the preprint in perpetuity. It is made available under aCC-BY 4.0 International license.

**CFY4881**	SEY6210.1 *Sec7-6xDsRed::URA3::ura3* *leu2::Age2 (I129 - S298)-Neon-3xFLAG::LEU2*	This study
**CFY4898**	SEY6210.1 *age2Δ::KanMX* *Sec7-6xDsRed::URA3* *leu2::Age2(SIL231NFF)-Neon-3xFLAG::LEU2*	This study
**CFY4899**	SEY6210.1 *age2Δ::KanMX* *Sec7-6xDsRed::URA3* *leu2::Age2(SILSLY231NFFNFF)-Neon-3xFLAG::LEU2*	This study
**CUY4655**	W303 *tor1-1* *fpr1::NatMX* *Pma1-2xFKBP12::TRP1*	(Auffarth et al., 2014)

bioRxiv preprint doi: https://doi.org/10.1101/2023.07.23.550229; this version posted July 24, 2023. The copyright holder for this preprint (which was not certified by peer review) is the author/funder, who has granted bioRxiv a license to display the preprint in perpetuity. It is made available under aCC-BY 4.0 International license.

Table S2. Plasmids used in this study:

Name	Description	Vector Backbone	Source
**pRS305**	Integration vector (LEU2)	pRS305	(Sikorski & Hieter, 1989)
**pRS414**	Vector (TRP1)	pRS414	(Sikorski & Hieter, 1989)
**pRS415**	Vector (LEU2)	pRS415	(Sikorski & Hieter, 1989)
**pArf1**	Arf1 (Expression plasmid)	pET3	(Weiss et al., 1989)
**pNmt1**	Nmt1 (Expression plasmid)	pCYC	(Duronio et al., 1990)
**pLT1**	FRB-Mars-Arf1(Q71L)ΔN13 (GPD promoter, CYC1 terminator)	pRS423	(Thomas et al., 2021)
**CFB3518**	Arf1-GFP	pRS415	B. Brownfield
**pCF1022**	Arf1	pRS416	(Thomas & Fromme, 2016)
**pCF1023**	Arf1	pRS415	(Thomas et al., 2021)
**pCF1053**	ΔN17-Arf1 (Expression plasmid)	pET28a	(Richardson et al., 2012; Richardson & Fromme, 2015)
**pCF1066**	Age2	pRS416	This study
**pKM38**	Arf1-GSSG-TetCys	pRS415	This study
**pKM55**	4xGga1$^{\text{N-GAT}}$-Neon (Integration plasmid)	pRS305	This study
**pKM73**	4xGga1$^{\text{N-GAT}}$-Neon (Expression plasmid)	pGEX-6P-1	This study
**pKM90**	Age2-Neon (ADH1 terminator)	pRS415	This study
**pKM92**	Age2-Neon-3xFLAG (Integration plasmid)	pRS305	This study
**pKM93**	Age2 (S231A)-Neon-3xFLAG (Integration plasmid)	pRS305	This study
**pKM94**	Age2 (I232A)-Neon-3xFLAG (Integration plasmid)	pRS305	This study
**pKM95**	Age2 (L233A)-Neon-3xFLAG (Integration plasmid)	pRS305	This study
**pKM96**	Age2 (S234A)-Neon-3xFLAG (Integration plasmid)	pRS305	This study

bioRxiv preprint doi: https://doi.org/10.1101/2023.07.23.550229; this version posted July 24, 2023. The copyright holder for this preprint (which was not certified by peer review) is the author/funder, who has granted bioRxiv a license to display the preprint in perpetuity. It is made available under aCC-BY 4.0 International license.

**pKM97**	Age2 (L235A)-Neon-3xFLAG (Integration plasmid)	pRS305	This study
**pKM98**	Age2 (Y236A)-Neon-3xFLAG (Integration plasmid)	pRS305	This study
**pKM99**	Age2 (KK229AA)-Neon-3xFLAG (Integration plasmid)	pRS305	This study
**pKM127**	Age2 (R52K)-Neon-3xFLAG (Integration plasmid)	pRS305	This study
**pKM131**	Age2 (M1-P239)-Neon-3xFLAG (Integration plasmid)	pRS305	This study
**pKM132**	Age2(M1-P150)-GS-Age2(G223-S298)-Neon-3xFLAG (Integration plasmid)	pRS305	This study
**pKM133**	Age2 (KKSILSLY 229 AAAAAAAA)-Neon-3xFLAG (Integration plasmid)	pRS305	This study
**pKM135**	GST [PreScission] Age2 (GAL1 promoter)	pYC230	This study
**pKM136**	Age2 (I129 - S298)-Neon-3xFLAG (Integration plasmid)	pRS305	This study
**pKM145**	Age2(M1-P150)-GS(72aa)-Age2(G223-S298)-Neon-3xFLAG (Integration plasmid)	pRS305	This study
**pKM146**	Age1-Neon (ADH1 promoter, CYC1 terminator)	pRS414	This study
**pKM152**	GST [PreScission] Age2 (KKSILSLY 229 AAAAAAAA) (GAL1 promoter)	pYC230	This study
**pKM154**	Age2 AH (RNDLKKSILSLYSK) (2X)-Neon (YPT1 promoter and terminator)	pRS415	This study
**pKM156**	Age2 (SIL231NFF)-Neon-3xFLAG (Integration plasmid)	pRS305	This study
**pKM157**	Age2 (SILSLY231NFFNFF)-Neon-3xFLAG (Integration plasmid)	pRS305	This study

All plasmids (besides plasmids for protein expression) express genes under their endogenous promoter and terminator unless otherwise denoted.

bioRxiv preprint doi: https://doi.org/10.1101/2023.07.23.550229; this version posted July 24, 2023. The copyright holder for this preprint (which was not certified by peer review) is the author/funder, who has granted bioRxiv a license to display the preprint in perpetuity. It is made available under aCC-BY 4.0 International license.

Table S3. Compositions of liposomes used in this study

Lipid	TGN	Folch
DOPC	24	-
POPC	6	-
DOPE	7	-
POPE	3	-
DOPS	1	-
POPS	2	-
DOPA	1	-
POPA	2	-
PI	29	-
PI(4)P	1	-
CDP-DAG	2	-
PO-DAG	4	-
DO-DAG	2	-
Ceramide (C18)	5	-
Cholesterol	10	-
Folch Lipids (Folch fraction I)	-	99
DiR Dye	1	1
